# Left ventricular geometry predicts ventricular tachyarrhythmia in patients with left ventricular systolic dysfunction: a comprehensive cardiovascular magnetic resonance study

**DOI:** 10.1186/s12968-017-0396-9

**Published:** 2017-10-23

**Authors:** Shiro Nakamori, Haisam Ismail, Long H. Ngo, Warren J. Manning, Reza Nezafat

**Affiliations:** 1 Department of Medicine, Cardiovascular Division, Beth Israel Deaconess Medical Center and Harvard Medical School, 330 Brookline Avenue, Boston, MA 02215 USA; 2 Radiology, Beth Israel Deaconess Medical Center and Harvard Medical School, Boston, MA USA

**Keywords:** Cardiomyopathies, Cardiovascular magnetic resonance, Implanted cardioverter-defibrillator, Left ventricular ejection fraction, Primary prevention, Sphericity index, Ventricular arrhythmia

## Abstract

**Background:**

Most patients with implantable cardioverter-defibrillator (ICD) implantation fail to utilize the device resulting in increasing societal costs and patient exposure to device morbidity. We sought to determine whether volumetric cardiovascular magnetic resonance (CMR) left ventricular (LV) spherical remodeling predicts future ventricular arrhythmias in primary ICD patients with reduced LV ejection fraction (EF).

**Methods:**

Sixty-eight consecutive patients with transthoracic echocardiographic LVEF <35% referred for CMR prior to ICD implantation for primary prevention of sudden death were identified. Sphericity index was measured as the ratio of LV end-diastolic volume (from cine short axis stack) to the volume of a sphere with a LV end-diastolic 4-chamber length diameter.

**Results:**

During a median follow-up of 55 months (interquartile range; 28–88), 15 patients (22%) received appropriate ICD therapy. Multivariable Cox’s proportional hazard modeling identified increased CMR-derived sphericity index as the strongest independent predictor of appropriate ICD therapy (hazard ratio [HR], 1.09; 95% confidence interval [CI], 1.02 to 1.16; *p* = 0.007). In addition, dichotomized volumetric CMR-derived sphericity index ≥0.57 carried a 4-fold hazard risk for appropriate ICD therapy, controlling for age and LVEF (HR, 4.49; 95% CI, 1.53 to 13.21; *p* = 0.006). When sphericity index, LVEF and mass index were used in combination, important incremental prognostic information was achieved (net reclassification improvement, 0.42; 95% CI, 0.06 to 0.77).

**Conclusions:**

The combined assessment of LV geometry, mass index and systolic function may provide incremental prognostic information regarding ventricular arrhythmia requiring appropriate ICD therapy in primary prevention patients with reduced LVEF.

## Background

The implanted cardioverter-defibrillator (ICD) is an established therapy for reducing mortality in patients with life-threating ventricular arrhythmia (VA) [1, 2]. Current guidelines for primary prevention ICD includes symptoms of heart failure and reduced left ventricular (LV) ejection fraction (LVEF) [3–5], but only a small percentage of primary prevention ICD recipients actually receive appropriate ICD therapy [6, 7] resulting in increasing societal costs and patient morbidity. If ICD therapy is to be used in a more cost-effective and lower morbidity manner, identifications of variables more predictive of appropriate ICD therapy are needed.

Late gadolinium enhancement (LGE) cardiovascular magnetic resonance (CMR) is the gold standard for the assessment of regional myocardial fibrosis and may help predict VA and sudden cardiac death (SCD) [8–12]. Heterogeneous LGE scar but not LGE volume is predictive for VA [10], but reproducible measurement of heterogeneous LGE scar is difficult [13]. Although most CMR studies give the highest priority to the assessment of LGE scar tissue characteristics, 2D transthoracic echocardiographic adverse LV remodeling, as well as LV relative wall thickness are also associated with VA [14–17]. Cine CMR is accurate, reproducible, and widely considered the non-invasive gold standard for morphological and functional assessment of the LV. However, no data are currently available regarding the association between CMR-derived LV geometric parameters and VA risk. Accordingly, the purpose of this study was to evaluate whether easily derived LV geometry metrics provide additive predictive value for the prediction of future VA in patients with reduced LVEF receiving primary prevention ICD therapy.

## Methods

### Study population

We retrospectively identified 71 consecutive patients who had undergone ICD implantation for primary SCD prevention who had a comprehensive CMR study before ICD implantation. Subjects were identified by querying the Beth Israel Deaconess Medical Center clinical CMR and ICD databases from April 2004 to December 2014. Exclusion criteria were: 1) idiopathic outflow tract ventricular tachycardias, 2) Brugada, and Long QT syndromes, 3) hypertrophic, inflammatory, infiltrative, and arrhythmogenic cardiomyopathies. Ischemic etiology was defined as the presence of any epicardial coronary artery dimeter stenosis >70%, a history of myocardial infarction, or a subendocardial based LGE pattern. Patient demographics and clinical follow-up records from the hospital electronic medical records were reviewed. The study was carried out with Beth Israel Deaconess Medical Center Institutional Review Board approval which waived written informed consent.

### Image acquisition

All CMR images were acquired on a 1.5 Tesla scanner (Achieva 1.5 T, Philips Medical Systems, Best, Netherlands) equipped with a 5-element or 32-element cardiac coil. The CMR protocol included cine and LGE. To assess LV/right ventricular (RV) myocardial function, geometry and mass, 10 to 12 short-axis stack cine images and 4-chamber long axis image were acquired using a cine balanced steady state free precession sequence (slice thickness, 8-mm; gap, 2-mm, in-plane spatial resolution 2 × 2 mm, 30 ms temporal resolution) [18]. Ten to 20 min after injection of 0.1–0.2 mmol/kg of Gd-DTPA (Magnevist; Bayer Schering, Berlin, Germany) or Gd-BOPTA (MultiHance; Bracco Imaging SpA, Milan, Italy), short- and long-axis 2D inversion recovery LGE images were acquired using a breath-hold, segmented inversion-recovery sequence (8-mm slice thickness, 2-mm inter-slice gap, TR, 4.2 ms; TE, 1.8 ms; FA, 20°; FOV, 320 × 320 mm^2^; matrix, 160 × 160; and spatial resolution of 2 mm^2^). In 35 patients, LGE was performed using a 3D phase sensitive inversion recovery sequence (PSIR) (5-mm slice thickness, TR, 4.2 ms; TE, 1.8 ms; FA, 15°; FOV, 320 × 320 mm^2^; acquisition matrix, 176 × 156; and spatial resolution,1.8 × 2.0 mm^2^).

### Image analysis

**Fig. 1:**
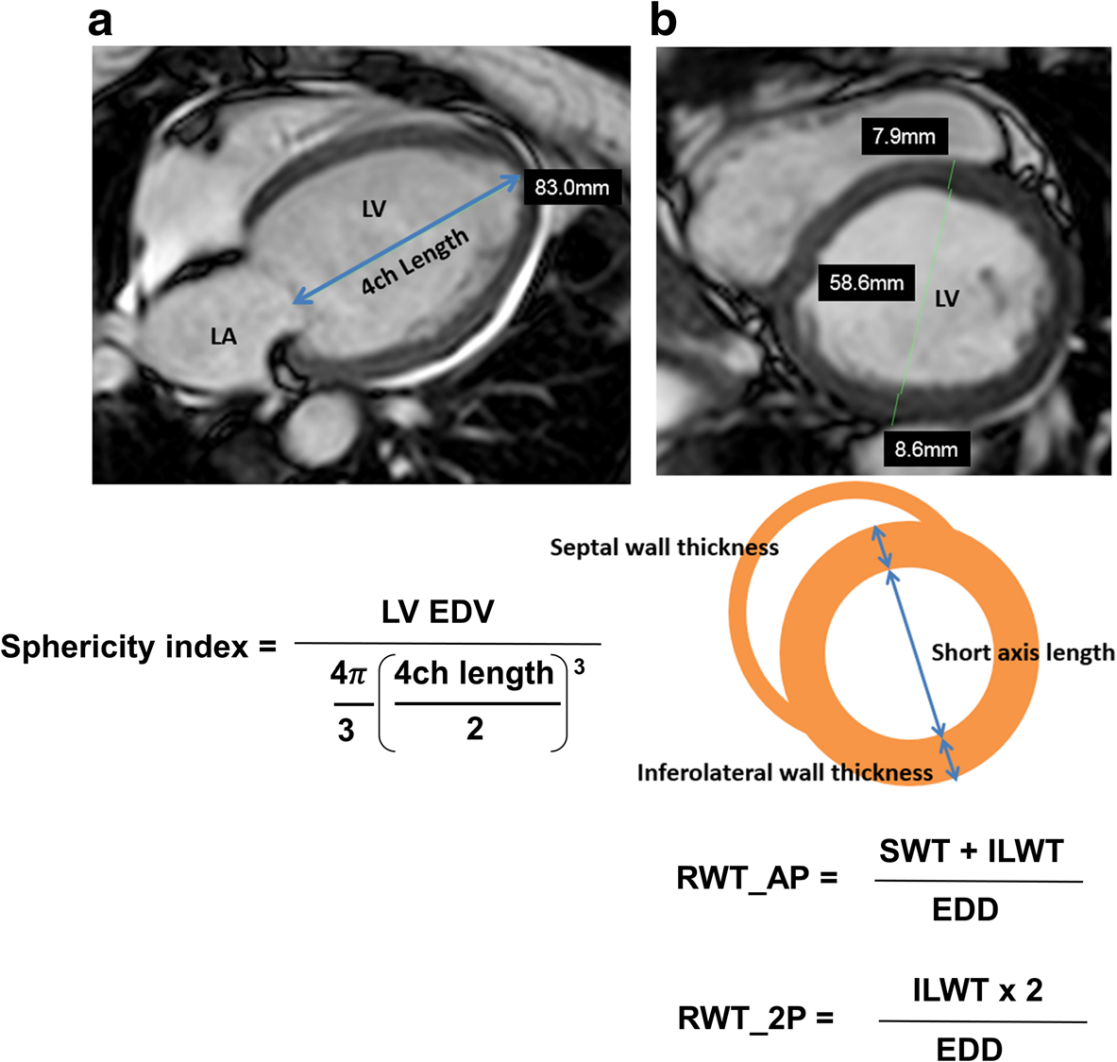
Measurements of sphericity index and relative wall thickness. Determination of (**a**) left ventricular (LV) sphericity index; LV end-diastolic volume (EDV), derived by cine short axis cardiovascular magnetic resonance, is divided by volume of a sphere with diameter equal to LV 4ch end-diastolic length. **b** relative wall thickness (RWT); LV anteroseptal wall thickness (SWT) plus inferolateral wall thickness (ILWT) is divided by LV end-diastolic diameter (EDD) from the short-axis slice immediately basal to the papillary muscles. RWT_2 is calculated as 2 × ILWT divided by LV EDD

CMR images were analyzed by a investigator blinded to ICD therapy using commercial workstations (Extend MR WorkSpace, version 2.3.6.3, Philips Healthcare, OsiriX environment, Pixmeo, Geneva, Switzerland). At end-diastole and end-systole, epi- and endocardial LV borders were manually traced from contiguous short-axis cine images covering the LV apex to mitral valve plane to calculate LV and RV end-diastolic volume (EDV) and end-systolic volume, stroke volume, and ejection fraction (EF). LV mass was calculated as the sum of the myocardial volume multiplied by the specific gravity (1.05 g/mL) of myocardial tissue. Sphericity index was calculated as the ratio of the LV EDV to the volume of a sphere with the diameter of the LV end-diastolic long axis from a 4-chamber cine image (=LV volume/[LV long axis length [3]×π/6]) [19, 20]. Relative wall thickness (RWT) was computed as the ratio of LV anteroseptal plus inferolateral wall thickness to end-diastolic cavity dimension measured at the slice immediately basal to the papillary muscles [21]. RWT_2 was also calculated as 2 times inferolateral wall divided LV end-diastolic diameter (Fig. 1). On LGE images, the presence or absence of LGE was visually assessed. LGE volume was assessed using a custom software developed in Matlab (MathWorks, Natick, Massachusetts, USA), which enables manual segmentation of scarred myocardium and normal remote region and quantifies LGE region using thresholding techniques. For each short-axis cross section, after the endocardial and epicardial borders were traced, a region-of-interest was defined in the normal remote myocardium without any artifact. The software calculated mean and standard deviation (SD) of remote region signal intensity (SI) and thresholds of all pixels with SI greater than mean + 2, 4, or 6SD of remote region, and reported a total LGE volume. Heterogeneous scar was defined as the difference between 2SD and 4SD (2-4SD), between 4SD and 6SD (4-6SD), and between 2SD and 6SD (2-6SD).

### Follow-up

Patients were implanted with a conventional or a biventricular ICD device at the discretion of the implanting physician and without knowledge of sphericity index, RWT or RWT_2. All devices were programmed for both anti-tachycardia pacing and shock with three zones of therapy including shock for ventricular fibrillation (VF), anti-tachycardia pacing followed by shock for fast ventricular tachycardia (VT), and a monitored zone for slower VT. Exact therapy settings were adjusted at the discretion of the implanting physician. Devices were interrogated at 1 and 3 months after implantation and every 6 months thereafter in the Device Clinic, during which the device was interrogated, and adjudication of stored ICD electrograms was performed by an electrophysiologist blinded to CMR findings (HI). The primary end point for our study was the delivery of appropriate ICD therapy for VT, VF, or sustained VT > 30-s duration as documented by the device and recorded in the patient’s online medical record.

### Statistical analysis

Statistical analyses were performed using SPSS (v19, International Business Machines, Inc., Armonk, New York, USA) and R version 3.2.3 (R Project for Statistical Computing). Continuous variables are expressed as mean ± standard deviation (SD) or median [quartiles] as appropriate, and compared using an unpaired Student’s t-test or Mann-Whitney nonparametric test if not normally distributed. Categorical variables were reported as counts and percentages, and compared using a chi-square test. To compare the relationship between the sphericity index and LVEF, a regression analysis was performed, and an analysis of covariance was used to test for equality of the regression slopes between patients with and without appropriate ICD therapy. All tests were 2 sided and *p* value <0.05 considered significant. Kaplan-Meier curves were used to estimate the distribution of time to the first episode of appropriate ICD therapy or sustained VT. Differences between time-to-event curves were compared with the log-rank test. Univariable Cox regression models were used to assess the association between each variable and the primary end point. For multivariable modeling, using the rule of thumb of having between 5 and 10 outcomes per predictor (we had 15 with appropriate ICD therapy), we decided to include 2 most significant variables from the univariable models (LVEF and sphericity index) and 1 covariate (age). We then used stepwise-forward selection which yielded only sphericity index in the final model. From this final model, we then searched for the optimal threshold of sphericity index (0.57) that yielded the largest area under the receiver operating characteristic (ROC) curve. Using this threshold, we then dichotomized the scale of sphericity index into binary and assessed the associations of sphericity index ≥0.57 to appropriate ICD therapy adjusted to age and LVEF. All reported associations in this study are hazard ratios (HR) and their corresponding 95% confidence intervals (CI). Reclassification of patients was determined using net reclassification improvement analysis for appropriate ICD therapy and obtained by adding sphericity index and LV mass index (LVMI) status to the model based on LVEF. Because no conventional cut-off values exist for the onset of ICD therapy in such population, risk categories were used to stratify patients into low-risk (0% to <10%), intermediate risk (10% to <20%) and high-risk (≥20%) categories. Categorical net reclassification improvement was computed together with integrated discrimination improvement.

## Results

### Patient population

Of the 71 identified patients, 3 patients (4%) were lost to follow-up and were excluded, leaving 68 patients for the final analysis. Their clinical and CMR characteristics are summarized in Tables 1 and 2. The majority were men (74%) with almost half (46%) having an ischemic cardiomyopathy and one third received a biventricular ICD. Sixty-six patients (97%) had LV dilatation (LVEDV index ≥95 ml/m^2^ in men *i* ≥ 78 ml/m^2^ in female). Sphericity index moderately and positively correlated with LVEDVI (*r* = 0.30, *p* = 0.02) and negatively correlated with LVEF (*r* = −0.45, *p* < 0.001), but did not correlate with LVMI (*r* = 0.17, *p* = 0.17).

**Table 1:** Patients clinical characteristics

Characteristics	All patients	No appropriate ICD therapy	Appropriate ICD therapy	*P*-value
	(*n* = 68)	(*n* = 53)	(*n* = 15)	
Age, yrs	63 ± 12	63 ± 12	66 ± 10	0.38
Male gender, n (%)	50 (74)	37 (70)	13 (87)	0.19
Ischemic cardiomyopathy (%)	31 (46)	21 (40)	10 (67)	0.06
Received biventricular ICD, n (%)	23 (34)	19 (36)	4 (27)	0.51
BSA, m^2^	1.95 ± 0.25	1.94 ± 0.25	1.98 ± 0.23	0.64
Hypertension (%)	51 (75)	38 (72)	13 (87)	0.24
Diabetes mellitus (%)	21 (31)	15 (28)	6 (40)	0.39
Dyslipidemia (%)	42 (62)	33 (62)	9 (60)	0.87
Serum creatinine, mg/dl	1.09 ± 0.25	1.06 ± 0.25	1.17 ± 0.21	0.16
QRS duration, ms	125 ± 31	123 ± 33	131 ± 24	0.39
NYHA functional class, n (%)				0.05
I	3 (4)	2 (4)	1 (7)	
II	25 (37)	23 (43)	2 (13)	
III	37 (55)	26 (49)	11 (73)	
IV	3 (4)	2 (4)	1 (7)	
Medication use, n (%)				
ACEI or ARB	62 (91)	47 (89)	15 (100)	0.17
Beta-blocker	59 (87)	46 (87)	13 (87)	0.99
Antiarrhythmics	6 (9)	5 (9)	1 (7)	0.74
Antiplatelet agents	53 (78)	39 (74)	14 (93)	0.10
Diuretics	19 (28)	15 (28)	4 (27)	0.90
Length of follow-up, months	58 ± 34	57 ± 36	59 ± 39	0.83

*ACEI* angiotensin converting enzyme inhibitor, *ARB* angiotensin receptor blockers, *BSA* body surface area, *ICD* implantable cardioverter-defibrillator, *NYHA* New York Heart Association, Values in parentheses represent percents

**Table 2:** Patients CMR characteristics

Characteristics	All patients	No appropriate ICD therapy	Appropriate ICD therapy	*P*-value
	(*n* = 68)	(*n* = 53)	(*n* = 15)	
LVDd (SAX), mm	67.6 ± 6.7	67.3 ± 6.6	68.7 ± 7.4	0.45
LV length (4 chamber), mm	97.8 ± 8.9	98.2 ± 9.5	96.1 ± 6.7	0.45
Sphericity index	0.54 ± 0.08	0.52 ± 0.07	0.60 ± 0.08	0.001
Septal wall thickness, mm	8.6 ± 2.6	8.4 ± 2.4	9.1 ± 3.1	0.39
Inferolateral wall thickness, mm	7.4 ± 2.0	7.4 ± 1.9	7.5 ± 2.6	0.84
RWT_2P	0.22 ± 0.06	0.22 ± 0.06	0.22 ± 0.08	0.99
RWT_AP	0.24 ± 0.06	0.24 ± 0.06	0.24 ± 0.08	0.77
LV EDV, ml	268.6 ± 80.3	261.6 ± 76.8	293.6 ± 89.9	0.17
LV EDVI, ml/m2	137.5 ± 36.0	134.0 ± 31.7	149.6 ± 47.5	0.14
LV ESV, ml	192.5 ± 78.0	182.2 ± 71.9	228.9 ± 89.8	0.04
LV EF, %	30.1 ± 9.4	31.8 ± 8.9	23.9 ± 8.7	0.003
LV mass, g	155.3 ± 46.4	147.5 ± 40.3	182.7 ± 57.0	0.009
LV mass index, g/m2	80.1 ± 22.6	76.5 ± 19.5	92.6 ± 28.5	0.014
LV mass/LV EDV, g/ml	0.59 ± 0.13	0.58 ± 0.13	0.63 ± 0.13	0.16
LV LGE, n (%)	38 (56)	27 (51)	11 (73)	0.11
LV LGE2SD, g	26.9 ± 33.5	23.8 ± 31.9	38.0 ± 38.2	0.13
LV LGE4SD, g	20.9 ± 29.1	19.1 ± 28.9	27.4 ± 30.1	0.18
LV LGE6SD, g	16.2 ± 24.5	15.1 ± 24.7	20.2 ± 24.5	0.17
Heterogeneous LGE(2-4SD), g	6.6 ± 9.8	5.5 ± 8.6	10.6 ± 12.8	0.09
Heterogeneous LGE(4-6SD), g	4.7 ± 6.8	4.0 ± 6.0	7.1 ± 8.9	0.10
Heterogeneous LGE(2-6SD), g	11.3 ± 15.3	9.5 ± 13.0	17.8 ± 20.8	0.12
RV EDV, ml	156.6 ± 58.5	156.4 ± 59.6	157.3 ± 56.5	0.96
RV EDVI, ml/m2	77.9 ± 19.9	77.5 ± 18.3	79.2 ± 25.4	0.77
RV ESV, ml	80.9 ± 42.0	77.5 ± 38.2	92.9 ± 53.0	0.21
RV EF, %	48.9 ± 12.5	50.3 ± 12.0	43.8 ± 13.3	0.08
RV EF < 40, n (%)	17 (25)	9 (17)	8 (53)	0.02

Values in parentheses represent percents

*Dd* diastolic dimension, *EDV* end-diastolic volume, *EDVI* end-diastolic volume index, *EF* ejection fraction, *ESV* end-systolic volume, *ICD* implantable cardioverter-defibrillator, *LGE* late gadolinium enhancement, *LV* left ventricular, *RV* right ventricular, *RWT* relative wall thickness, *SAX* short axis transection, *SD* standard deviation

**Fig. 2:**
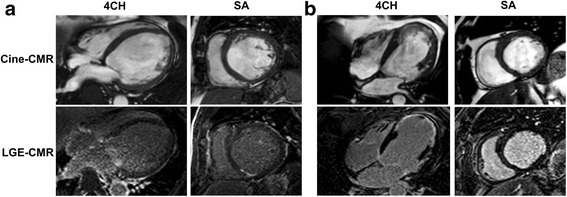
Representative ischemic cardiomyopathy cases with and without increased sphericity index. Example cases (**a**) 68 year-old man with prior inferior myocardial infarction who received appropriate ICD therapy. Larger sphericity index of 0.73 as well as severe LV dysfunction (LVEF = 18%) was documented although LGE-CMR image showed only a small area of focal subendocardial inferior wall enhancement. **b** 61 year-old man with an extensive anteroseptal myocardial infarction who did not receive ICD therapy. Sphericity index was 0.48, however there is marked dyskinesis with LVEF 21% on the LV apex and transmural LGE were observed

**Fig. 3:**
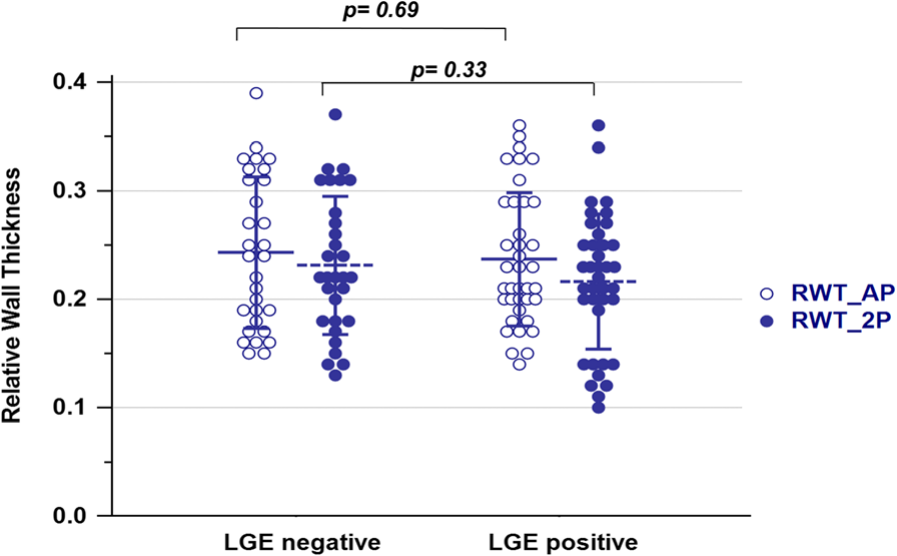
Individual subject relative wall thickness data of patients with and without LGE. There were no significant differences in relative wall thickness (RWT_AP and RWT_2P) between LGE positive vs. LGE negative group

During a median follow-up of 55 months (interquartile range; 28–88), 15 patients (22%) received appropriate ICD therapy. Appropriate ICD therapies were delivered in 11/45 (24%) with conventional ICDs and 4/23 (17%) with biventricular ICDs (*p* = 0.51). There was a trend for patients with an ischemic cardiomyopathy to receive appropriate ICD therapy (32% vs 14%, *p* = 0.06). Patients with and without appropriate ICD therapy had similar baseline age, gender, history of hypertension, diabetes mellitus and dyslipidemia (all p = NS), while patients that received appropriate ICD therapy tended to have a *higher* NYHA functional class (*p* = 0.05). There was a trend for higher LV end-systolic volume and lower LVEF in patients with appropriate ICD therapy (*p* = 0.04 and 0.003, respectively). Patients with ICD therapy had significantly lower RVEF (*p* = 0.02). LVMI was significantly higher in patients with appropriate ICD therapy compared with that in patients without ICD therapy (*p* = 0.01). In addition, LV sphericity index was significantly higher in patients with appropriate ICD therapy (*p* = 0.001). Figure 2 shows representative cases from ischemic cardiomyopathy with and without increased sphericity index. The presence and any extent of LGE using different thresholding (2SD, 4SD, and 6SD) were not associated with receiving appropriate ICD therapy (*p* = 0.11, 0.13, 0.18 and 0.17, respectively). There was a trend for heterogeneous scar (2-4SD) to more likely receive appropriate ICD therapy (*p* = 0.09). RWT was not associated with the presence of LGE (Fig. 3).

### LV geometry and appropriate ICD therapy

**Fig. 4:**
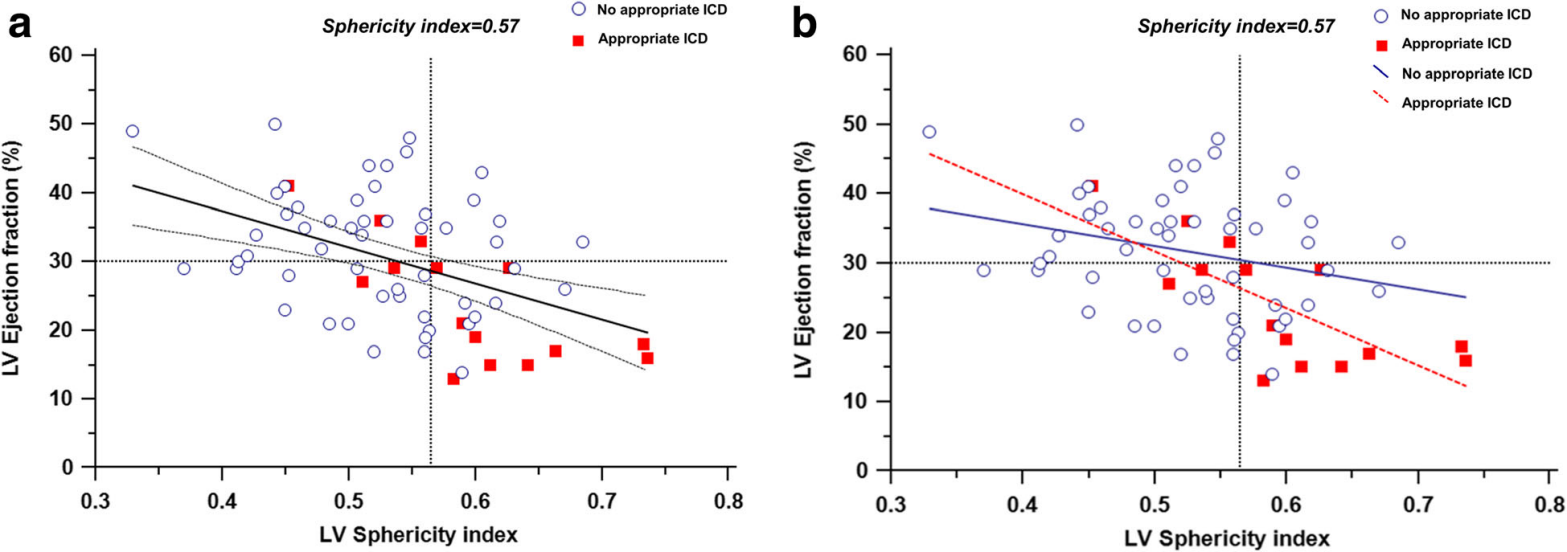
Correlation between LV EF and sphericity index. **a** Sphericity index moderately and negatively correlated with LV EF in the whole population (*n* = 68) (*r* = −0.45, *P* < 0.001). **b** In the subgroup analysis of patients with and without appropriate ICD therapy, sphericity index significantly correlated with LVEF in patients *with* appropriate ICD therapy alone (*r* = −0.74, *P* = 0.002) while there was no significant association between sphericity index and LVEF in patients without appropriate ICD therapy (*r* = −0.26, *P* = 0.06). The line for the appropriate ICD group lies significantly below the line for the no appropriate ICD group (*p* < 0.05)

**Fig. 5:**
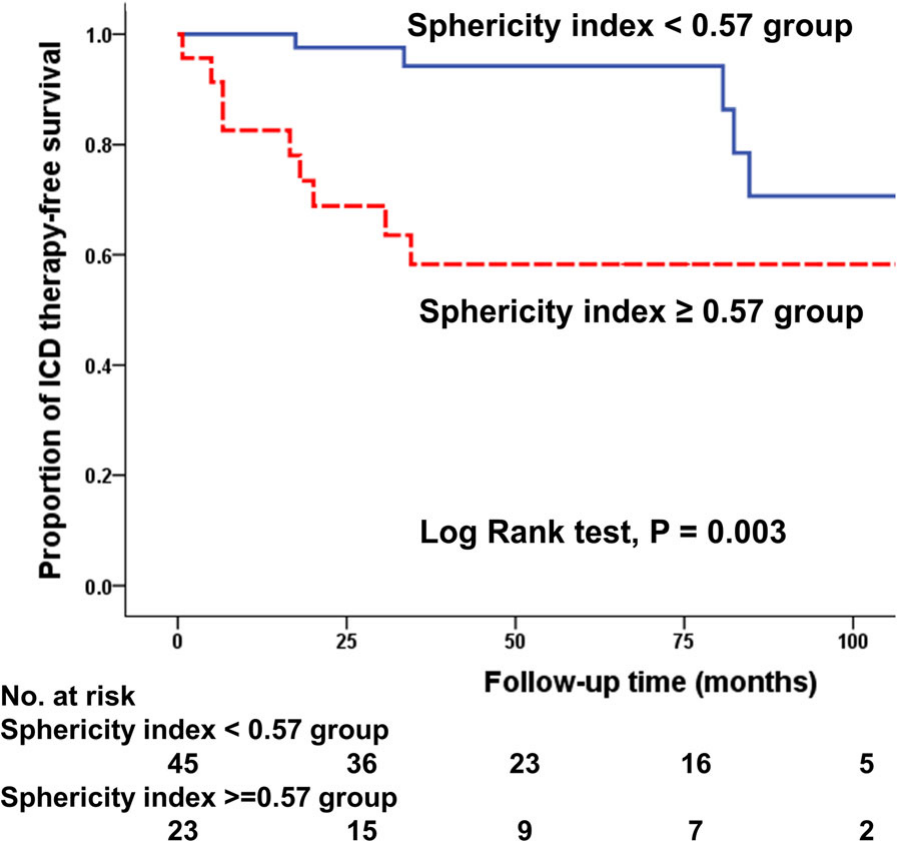
Survival curve for ventricular arrhythmic events requiring ICD therapy using the sphericity index threshold of 0.57

Univariable and multivariable analyses of clinical and CMR parameters for appropriate ICD therapy are summarized in Table 3. In multivariable analysis, increased LV sphericity index was the strongest independent predictor of appropriate ICD therapy (hazard ratio [HR]; 1.09; 95% confidence interval [CI] 1.02 to 1.16, *p* = 0.007). Although reduced LVEF was an important predictor of appropriate ICD therapy in univariate analysis, LVEF was no longer significant in multivariate analysis because of significant negative correlation with sphericity index (*r* = −0.45, *p* < 0.001) (Fig. 4). The ROC curves showed a sphericity index of ≥0.57 to be the optimal cut-off points for appropriate ICD therapy, with 67% sensitivity, 76% specificity and area under the ROC curve of 0.75 (95% CI 0.61 to 0.89). A sphericity index of ≥0.57 identified patients with a 4-fold hazard risk for appropriate ICD therapy, after adjusting for age and LVEF. Kaplan-Meier curves showed significantly lower ICD therapy-free survival in patients with sphericity index values ≥0.57 (*p* = 0.003) (Fig. 5). The c-statistic of LVEF and LVMI for predicting appropriate ICD therapy were 0.74 (95% CI 0.59 to 0.89) and 0.68 (95% CI 0.52 to 0.84), respectively. The optimal LVEF threshold of 30% determined by ROC curves provided 80% sensitivity and 57% specificity for appropriate ICD therapy. When sphericity index as well as LVMI, was combined with LVEF, we observed a greater c-statistic than either variable used individually (c-statistic = 0.81; 95% CI 0.66 to 0.95, Delong’ test; *p* = 0.13). Furthermore, the addition of sphericity index and LVMI values to LVEF yielded 4 correct (up) reclassifications and 2 incorrect (down) reclassifications in the 15 patients of receiving ICD therapy. Additionally, 19 correct (down) reclassifications and 4 incorrect (up) reclassifications occurred in the 53 patients who did not receive ICD therapy. Overall, the integration of sphericity index and LVMI values provided the improvement in risk stratification (net reclassification index (NRI) 0.42; 95% CI 0.06 to 0.77, *p* = 0.02). Reduced RVEF appeared more likely to predict appropriate ICD therapy (HR; 1.04, *p* = 0.054) and RVEF <40% (by dichotomous analysis) demonstrated a strong univariate association with appropriate ICD therapy (HR; 3.92; 95% CI 1.42 to 10.85, *p* = 0.009). However, RVEF did not significantly predict appropriate ICD therapy beyond LVEF.

**Table 3:** Univariable and multivariable Cox’s proportional hazard models for the association with appropriate ICD therapy

	Univariate analysis			Multivariate analysis model 1			Multivariate analysis model 2		
Characteristics	HR	95% CI	*P* Value	HR	95% CI	*P* Value	HR	95% CI	*P* Value
Age,yrs	1.01	0.96–1.06	0.63	1.02	0.97–1.08	0.41	1.02	0.97–1.08	0.43
Male	3.22	0.72–14.42	0.13						
Biventricular ICD	0.49	0.15–1.58	0.23						
Ischemic cardiomyopathy	2.42	0.83–7.10	0.11						
Hypertension	2.16	0.49–9.59	0.31						
Diabetes mellitus	2.04	0.72–5.78	0.18						
Dyslipidemia	0.77	0.27–2.24	0.63						
Serum creatinine, per 0.1 increase	1.19	0.97–1.46	0.10						
QRS duration	1.00	0.99–1.02	0.72						
NYHA ≥ III	2.65	0.75–9.42	0.13						
CMR parameters									
Sphericity index, per 0.01 increase	1.09	1.02–1.16	0.007	1.09	1.02–1.16	0.007			
Sphericity index > 0.57	4.49	1.53–13.21	0.006				4.49	1.53–13.21	0.006
RWT, per 0.01 increase	1.01	0.94–1.10	0.75						
LV EDVI	1.01	1.00–1.02	0.18						
LV ESV	1.01	1.00–1.01	0.039						
LV EF, per 1% decrement	1.09	1.03–1.15	0.005	1.07	0.99–1.13	0.08	1.07	0.99–1.13	0.08
LV mass index	1.01	1.00–1.02	0.039						
LV mass/LV EDV	4.56	0.11–198.25	0.43						
LV LGE	2.45	0.78–7.72	0.13						
LV LGE2SD	1.01	1.00–1.03	0.14						
LV heterogeneous LGE(2-4SD)	1.07	1.02–1.12	0.01						
RV EDVI	1.01	0.98–1.03	0.58						
RV ESV	1.01	1.00–1.02	0.10						
RV EF, per 1% decrement	1.04	1.00–1.08	0.054						
RV EF < 40%	3.92	1.42–10.85	0.009						

Variables given are mean ± SD or N (%) or median (interquartile range)

Abbreviation as in Tables 1 and 2

Model 1 included sphericity index, LVEF and age. Model 2 included sphericity index > 0.57, LVEF and age

HR (hazard ratio) refers to the ratio of hazards of the presence of the characteristic to the reference (absence), or to the change of 1 unit (continuous variable)

## Discussion

In this retrospective study of 68 consecutive patients referred for CMR prior to primary prevention ICD implantation, we demonstrate that: 1) CMR LV sphericity index is the strongest independent predictor of ventricular arrhythmias requiring appropriate ICD therapy, 2) CMR-derived sphericity index ≥0.57 is associated with a higher risk of appropriate ICD therapy adjusted for age, gender, and LVEF, 3) the integration of sphericity index to LV function assessment provides additional prognostic information regarding appropriate ICD therapy. Importantly, sphericity index can be easily derived and without a cost of additional scan time or need for gadolinium contrast.

Echocardiographic (echo)-derived LV sphericity is a marker of LV systolic dysfunction and exercise tolerance in heart failure [19, 22], an independent predictor of adverse cardiovascular events in ischemic [23] or non-ischemic cardiomyopathy [24], and is associated with increased LV wall stress [25]. Moreover, higher wall stress can alter the electrophysiologic properties through an increase in dispersion of action potential duration and membrane recovery [26, 27] and contribute as trigger for VA, although anatomical substrates for re-entrant VA have been mostly attributed to LV architectural changes, such as scar and interstitial fibrosis. Our study results are consistent with the recent study by Levine et al. showing that 2D transthoracic echo sphericity index predicts appropriate ICD therapy in patients with reduced LVEF [15]. However, selection of imaging planes and accuracy of 2D echo are dependent on operator experience, and limited acoustic windows may not allow accurate long axis views of the heart. In addition, biplane volumetric analysis by 2D echo depends on geometric assumptions and is subject to image-plane positioning errors. Thus, relatively small measurement errors can lead to some differences in sphericity index cut-off value between biplane 2D echo and volumetric CMR.

In a recent 2D echo study by Biton et al. [14], decreased RWT was associated with a higher risk of VA and cardiac death, leading to the conclusion that RWT is directly correlated with wall thickness and low RWT might mirror the extent of LV fibrosis/scarring. In contrast, the 2D echo results of the VALIANT study indicated higher RWT was related to an increased risk of cardiovascular death, MI and sudden death in post-MI population [28]. We found the impact of RWT had not been shown clearly, although patients with *increased* LVM/LVEDV appeared likely to receive appropriate ICD therapy. Furthermore, our observation was that the presence of LGE was *not* associated with regional wall thickness as well as RWT. This finding supports the results of Shah et al. who found myocardial thinning does not necessarily indicate scar tissue and might improve after revascularization in patients with coronary artery disease [29]. In ischemic cardiomyopathy, the extreme nonuniformity of the LV wall thickness in combination with marked variability in the extent or degree of adverse remodeling would be expected to contribute to considerable RWT overlap.

Our results showed the presence and extent of LGE was not a predictor for appropriate ICD therapy. Although focal scar evident by the presence and extent of LGE is known to be associated with increased risk of VA and ICD therapy [7, 8, 30–32], the electrophysiologic substrate for ventricular arrhythmias in patients with ischemic and non-ischemic cardiomyopathy is substantially different. Diffuse myocardial fibrosis, myocyte disarray, and membrane abnormalities likely form the substrate responsible for VA in patients with non-ischemic cardiomyopathy [33]. Therefore, heterogeneous patient population of ischemic and non-ischemic cardiomyopathy patients undergoing ICD implantation and LGE assessment may explain the inconsistency between the current study and previous studies of ischemic cardiomyopathy. Similar to our previous study [10], we also found that heterogeneous scar rather than presence and extent of LGE had univariate association with appropriate ICD therapy. These findings suggest that heterogeneous scar is a more sensitive marker of appropriate ICD therapy. Myocardial tissue characterization using T_1_ or extracellular volume (ECV) mapping allows for assessment of diffuse myocardial fibrosis. We have recently reported the native T_1_ is useful for predicting VA in non-ischemic cardiomyopathy [34]. Further studies are needed to confirm whether myocardial tissue characterization using newer technique, i.e. T_1_ or ECV mapping, provide incremental value to LGE and LV function/geometry for the prediction of future VA risk in patients with reduced LVEF receiving primary prevention ICD therapy.

Interestingly, we found RVEF <40% carried a 4-fold unadjusted hazard risk for appropriate ICD therapy, which implies a clinical importance for the accurate assessment of RV function by CMR. However, RV dysfunction was mainly related to indices of LV function and not associated with VA beyond LVEF and the presence of LGE. This finding may reflect the presence of other subclinical conditions, such as RV ischemia or post-capillary pulmonary hypertension that affect RV function and have negative impact on VA.

## Study limitations

Our study has several limitations. It is a retrospective study with a relatively small sample size, thus, the results should be interpreted cautiously. We studied patients referred for CMR prior to undergoing primary prevention ICD implantations spanning a period of 10 years. Heart failure management might have changed over the course of this follow-up period. We assessed CMR parameters at a single time. Changes in sphericity index may be more predictive of future VA than a single baseline measurement. Device choice and programming was not standardized and was left to the discretion of the operator.

## Conclusion

CMR-derived sphericity index may be an important predictor of VA and provide additive risk stratification for primary prevention ICD in patients with LV systolic dysfunction. Prospective, large multicenter studies are warranted to examine this easily obtained CMR parameter in the selection of patients for primary prevention ICD therapy.

## Abbreviations

CI: Confidence interval
CMR: Cardiovascular magnetic resonance
HR: Hazard ratio
ICD: Implantable cardioverter-defibrillator
LGE: Late gadolinium enhancement
LVEDV: Left ventricular end-diastolic volume
LVEF: Left ventricular ejection fraction
LVMI: Left ventricular mass index
ROC: Receiver operating characteristic
RVEF: Right ventricular ejection fraction
RWT: Relative wall thickness
VA: Ventricular arrhythmia
VF: Ventricular fibrillation
VT: Ventricular tachycardia
